# Frameshift variants in *C10orf71* cause dilated cardiomyopathy in human, mouse, and organoid models

**DOI:** 10.1172/JCI177172

**Published:** 2024-06-17

**Authors:** Yang Li, Ke Ma, Zhujun Dong, Shijuan Gao, Jing Zhang, Shan Huang, Jie Yang, Guangming Fang, Yujie Li, Xiaowei Li, Carrie Welch, Emily L. Griffin, Prema Ramaswamy, Zaheer Valivullah, Xiuying Liu, Jianzeng Dong, Dao Wen Wang, Du, Wendy K. Chung, Yulin Li

**Affiliations:** 1 Beijing Anzhen Hospital, Capital Medical University, Beijing, China.; 2 Beijing Institute of Heart, Lung & Blood Vessel Disease, Beijing, China.; 3 The Key Laboratory of Remodeling-Related Cardiovascular Diseases, Ministry of Education, Beijing, China.; 4 Novogene Co. Ltd., Beijing, China.; 5 Department of Cardiology, The First Affiliated Hospital of Zhengzhou University, Zhengzhou, China.; 6 Department of Pediatrics, Columbia University, New York, New York, USA.; 7 Maimonides Medical Center, New York, New York, USA.; 8 Broad Institute, Cambridge, Massachusetts, USA.; 9 Changping Laboratory, Beijing, China.; 10 Division of Cardiology, Tongji Hospital, Tongji Medical College, Huazhong University of Science and Technology, Wuhan, China.; 11 Boston Children’s Hospital, Harvard Medical School, Boston, Massachusetts, USA.

**Keywords:** Cardiology, Genetics, Cardiovascular disease, Genetic diseases, Genetic variation

## Abstract

Research advances over the past 30 years have confirmed a critical role for genetics in the etiology of dilated cardiomyopathies (DCMs). However, full knowledge of the genetic architecture of DCM remains incomplete. We identified candidate DCM causal gene, *C10orf71,* in a large family with 8 patients with DCM by whole-exome sequencing. Four loss-of-function variants of *C10orf71* were subsequently identified in an additional group of492 patients with sporadic DCM from 2 independent cohorts. C10orf71 was found to be an intrinsically disordered protein specifically expressed in cardiomyocytes. *C10orf71*-KO mice had abnormal heart morphogenesis during embryonic development and cardiac dysfunction as adults with altered expression and splicing of contractile cardiac genes. *C10orf71*-null cardiomyocytes exhibited impaired contractile function with unaffected sarcomere structure. Cardiomyocytes and heart organoids derived from human induced pluripotent stem cells with *C10orf71* frameshift variants also had contractile defects with normal electrophysiological activity. A rescue study using a cardiac myosin activator, omecamtiv mecarbil, restored contractile function in *C10orf71*-KO mice. These data support *C10orf71* as a causal gene for DCM by contributing to the contractile function of cardiomyocytes. Mutation-specific pathophysiology may suggest therapeutic targets and more individualized therapy.

## Abstract 2

The dilated cardiomyopathy gene *C10orf71* affects cardiac morphology and contractile function*.*

## Introduction

Cardiomyopathies are a heterogeneous group of cardiac disorders with primary abnormalities in the structure and function of the heart (1). These disorders are commonly divided into primary — including hypertrophic, dilated, arrhythmogenic right ventricular, and left ventricular noncompaction(LVNC) cardiomyopathy — and acquired — such as acute myocarditis, peripartum, and tachycardia-induced cardiomyopathy (2). Dilated cardiomyopathy (DCM) is the most common genetic cardiac disease, with a prevalence of 1 in 250 people worldwide (3). It is characterized by left or biventricular dilation, reduced systolic function, and, typically, autosomal dominant inheritance. Cardiomyopathies are common causes of heart failure and death and warrant prompt diagnosis and tailored treatment.

There is a critical role for genetics in the etiology of many cardiomyopathies. Hypertrophic cardiomyopathy and arrhythmogenic right ventricular cardiomyopathies are largely genetic diseases involving sarcomeric and desmosomal proteins, respectively (4). In contrast, DCM is a final common phenotype resulting from many proximal structural, physiological, or metabolic pathway derangements, making it the most genetically heterogeneous of all of the primary cardiomyopathies (5). More than 250 genes spanning over 10 gene ontologies have been implicated in DCM, including genes related to the sarcomere and Z disc, sarcoglycans, the cytoskeletal complex, transcription factors, calcium handling, the nuclear envelope, potassium and sodium channels, heat shock chaperones, and mitochondria (3). However, recent systematic review of evidence for DCM genes supports focusing on only 19 of these genes for routine clinical practice (6). The genes explain a minority of DCM cases, suggesting there are yet unidentified genetic contributors (7, 8). Identification of additional causal genes will not only aid in genetic diagnosis, but also reveal pathologic mechanisms of DCM, potentially illuminating therapeutic targets.

In the present study, we used exome sequencing to identify genetic causes of DCM in a large family with 8 patients with DCM. We then assessed additional patients with sporadic DCM and identified frameshift and stop gain variants in *C10orf71* as likely diagnoses. Detailed in vitro and in vivo functional experiments showed that *C10orf71* encodes an intrinsically disordered protein specifically expressed in cardiomyocytes (CMs) and contributing to contractile function.

## Results

### C10orf71 is a DCM causal gene.

**Figure 1:**
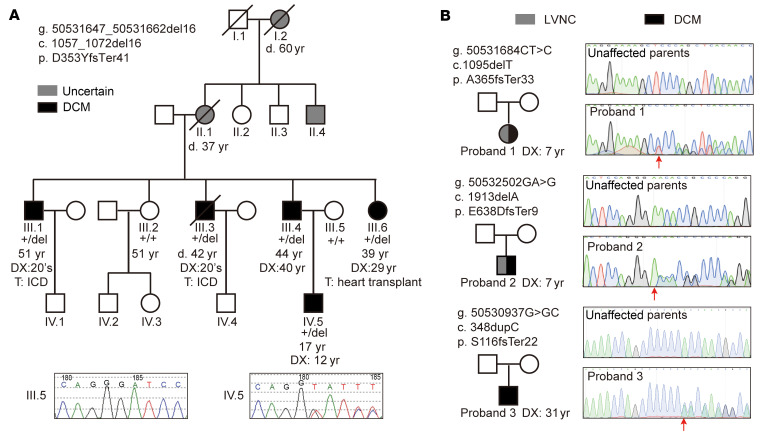
Frameshift mutations of *C10orf71* are associated with DCM. (**A**) Identification of a *C10orf71* variant segregating with DCM in a multigenerational family. d, death; DX, diagnosis; T, treatment; ICD, implantable cardioverter–defibrillator. (**B**) De novo *C10orf71* variants identified in 3 probands from Anzhen DCM cohort.

In a large DCM family at Columbia University Irving Medical Center (Figure 1A), the proband was a male who came to clinical attention at the age of 12 years old. He was found to have moderately diminished left ventricular systolic function with a ventricular internal diameter at end diastole (LVID__d__) of 6.23 cm (–2SD–2SD: 3.65–4.91) and left ventricular internal diameter at end systole (LVID__s__) of 4.79 cm (–2SD–2SD: 2.17–3.29) and a left ventricular ejection fraction (LVEF) of 45% on echocardiogram. His family history was marked for an extended multigenerational history of isolated DCM diagnosed in the 20–40-year age range in 5 family members (III.1, III.3, III.4, III.6, and IV.5). Additionally, 3 members in generation I and II (I.2, II.1, and II.4) were reported to have enlarged hearts by other family members. The paternal aunt of the proband (III.6) had a heart transplant at 29 years of age. The explanted heart weighed 420 grams. The ventricles were dilated and had endocardial thickening and interstitial fibrosis (Supplemental Figure 1; supplemental material available online with this article; https://doi.org/10.1172/JCI177172DS1). There was mild to moderate myocyte hypertrophy. The 2 paternal uncles of the proband (III.1 and III.3) were treated with implantable cardioverter–defibrillators (ICDs). Initial variant segregation analysis of whole exome sequencing (WES) for the proband, 4 affected family members (father, 2 paternal uncles, and a paternal aunt), and 2 unaffected members (mother and a paternal aunt) was performed in 2018 by GeneDx. No pathogenic variants in known DCM causal or candidate genes were identified. The *C10orf71* frameshift variant, NM_001135196.1: c.1057_1072del; p.(D353YfsTer41), was identified in all affected family members tested but absent in the unaffected members. Then, separate DNA samples were obtained from III.1–6 and IV.5, and the genotypes of the target site were confirmed with Sanger sequencing. The log odds (LOD) score for the association between genotype and phenotype in this family was 3.0. No other functional variant cosegregated with DCM in the large family.

We then screened for *C10orf71* loss of function (LOF) variants including stop gain, stop loss, frameshift insertion, and frameshift deletion in 2 independent sporadic DCM cohorts of Chinese ancestry. The Beijing Anzhen DCM cohort included 220 patients (Supplemental Table 1) with WES data. Among them, 106 patients were children and 114 were adults. We identified 3 frameshift variants (*C10orf71*: NM_001135196.1: c.1095delT, c.1913delA, and c.348dupC) carried by 3 patients who had no variant in known DCM causal genes and confirmed these variants with Sanger sequencing. We traced back and detected the genotype of their parents and found that the 3 variants were all de novo frameshift *C10orf71* variants (Figure 1B). Proband 1 and proband 2 were 7 years old at the time of diagnosis. Besides DCM, their echocardiograms also showed LVNC. Proband 1 had an LVID__d__ of 4.30 cm (–2SD–2SD: 3.05–4.28), LVID__s__ of 3.20 cm (–2SD–2SD: 1.79–2.87), and an LVEF of 50% (Table 1). Proband 2 had an LVID__d__ of 4.80 cm (–2SD–2SD: 3.11–4.35), LVID__s__ of 3.40 cm (–2SD–2SD: 1.81–2.93), and an LVEF of 50% (Table 1). Proband 3 was diagnosed with DCM at the age of 31, had an LVID__d__ of 6.90 cm (–2SD–2SD: 4.54–6.14), LVID_s_ of 6.10 cm (–2SD–2SD: 2.56–4.32), and an LVEF of 26%. There were no symptoms related to cardiomyopathy when he was younger. The Wuhan Tongji DCM cohort included 363 patients with WES data (9). Among them, 91 patients with deleterious variants in 33 known DCM genes were excluded from this study. We identified another heterogeneous stop gain variant (*C10orf71*: NM_001135196.1: c.G3053A) in 1 male patient diagnosed with DCM at the age of 38. He had an LVID_d_ of 6.50 cm, LVID_s_ of 5.00 cm, and an LVEF of 45% (Table 1). However, the phenotype and genotype information of his parents were not available.

*C10orf71* is located in 10q11.23. There is no known DCM causal gene located close to *C10orf71* (Supplemental Figure 2A). The gene closest to *C10orf71* is *DRGX*, approximately 36.6 Kbp away from *C10orf71*. To our knowledge, no study has reported a link between *DRGX* and DCM. We evaluated the linkage disequilibrium within 50 kb of either side of *C10orf71* and found that *C10orf71* was not in linkage disequilibrium with surrounding regions (Supplemental Figure 2B, the SNPs in the red box are located in *C10orf17*). Therefore, to our knowledge, there is no other gene in linkage disequilibrium with *C10orf71*, further supporting that *C10orf71* is a DCM causal gene.

All 5 predicted LOF variants were absent in gnomAD as well as over 1,000 in-house individuals of Chinese descent who acted as controls and had no clinical, electrocardiographic or echocardiographic evidence of cardiomyopathy. To directly confirm the effect of the variants on *C10orf71* expression, we performed site-directed mutagenesis in human *C10orf71* cDNA subcloned in the expression vector pCMV3 (Supplemental Figure 3A). Compared with WT plasmid, the mutant plasmids produced truncated protein products (Supplemental Figure 3B). We next detected whether the mRNA with the frameshift variant was degraded by nonsense-mediated mRNA decay (NMD). Because patient-derived CMs or human induced pluripotent stem cells (hiPSCs) were unavailable, we had to obtain CMs with endogenously expressed mutant C10orf71 by generating *C10orf71*-mutant hiPSCs. Using 2 hiPSC lines from individuals who were healthy (WT1 and WT2) (10, 11), we obtained 2 *C10orf71* mutant hiPSC lines (Mut1: c.173dupA and Mut2: c.172_182del, Supplemental Figure 4). Although the introduced mutations are different from those found in patients, they are also frameshift mutations located in the front of the only exon coding *C10orf71* protein, just like the mutations carried by patients. Therefore, detecting the mRNA levels of C10orf71 in the CMs derived from mutant hiPSCs can reflect whether the mRNA with frameshift variants in patient CMs is degraded by NMD. Using qPCR and transcriptome sequencing, we demonstrated that the level of frameshift variant mRNA was significantly decreased compared with that of the corresponding WT control (Supplemental Figure 5), suggesting that the frameshift variants acted as functional nulls.

### C10orf71 is a potential intrinsically disordered protein specifically expressed in CMs.

**Figure 2:**
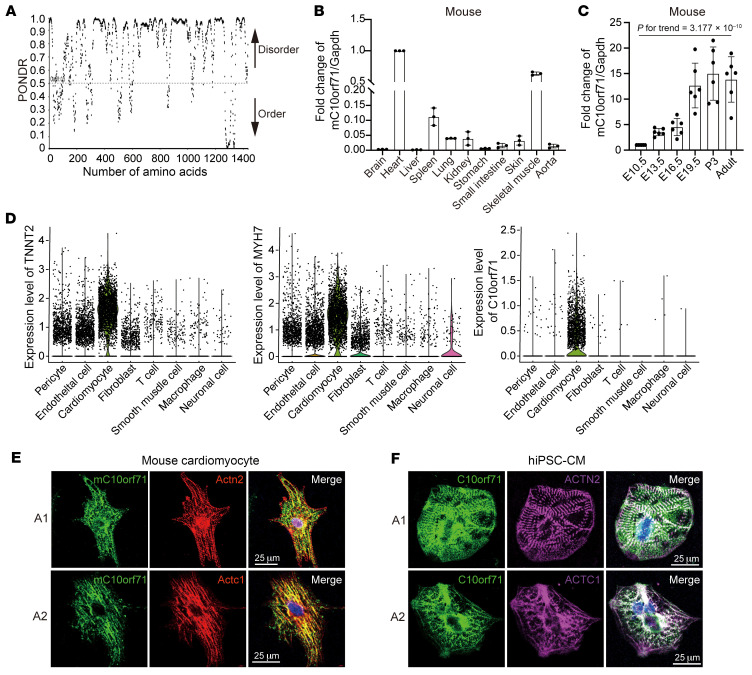
C10orf71 is an intrinsically disordered protein specifically expressed in CMs. (**A**) Result of folding prediction for the human full-length C10orf71 with PSIPRED. (**B**) Relative mRNA levels of m*C10orf71* in various adult mouse tissues; error bars indicate SD (*n* = 3). (**C**) Relative mRNA levels of m*C10orf71* during mouse heart development (*n* = 6). (**D**) *TNNT2*, *MYH7*, and *C10orf71* expression levels in each cell type of human heart. (**E** and **F**) Representative images showing double immunofluorescence staining of the CMs from C57BL/6 mice (**E**) and iPSCs (**F**). ACTN2 and ACTC1 are the markers for Z disc and myofibers, respectively. DAPI, nuclei stain, blue. A1/A2 indicates 2 independent duplicate samples. Scale bars: 25 μm.

Structural analysis showed that C10orf71 had almost no regular secondary structure and was in a disordered state throughout the entire length (Figure 2A and Supplemental Figure 6). AlphaFoldDB (https://alphafold.ebi.ac.uk/) also showed that most regions of C10orf71 had no fixed 3-dimensional protein structure (Supplemental Figure 7). In addition, C10orf71 fit to multiple characteristics of intrinsically disordered protein. For example, ProtScale showed that C10orf71 had high hydrophilicity (Supplemental Figure 8). The instability index of C10orf71 was computed to be 65.67 in ProtParam database, classifying the protein as unstable. These facts suggest that C10orf71 is an intrinsically disordered protein.

*C10orf71* was highly and specifically expressed in human heart and skeletal muscle (Supplemental Figure 9, from GTEx (V8)). The murine homologous gene, 3425401B19Rik, indicated as m*C10orf71* in this study, exhibited a similar expression pattern (Figure 2B). To determine the temporal expression pattern of m*C10orf71* in heart, we collected serial embryonic cardiac structures at 4 in utero stages (E10.5, E13.5, E16.5, and E19.5) and cardiac tissue at 3 days after birth from WT C57/BL6J mice. The expression of m*C10orf71* increased significantly during heart development (Figure 2C), like the cardiac marker *Tnnt2* (Supplemental Figure 10). This expression pattern was also found during myogenic differentiation (Supplemental Figure 11), suggesting that m*C10orf71* may be involved in the development and maturation of muscle. Using single nucleus RNA sequencing data of human hearts, we demonstrated that *C10orf71* is specifically expressed in CMs, even more specifically than well-established CM markers, such as *TNNT2* and *MYH7* (Figure 2D and Supplemental Figure 12). Published single-cell sequencing data (12) validated this specific expression pattern and further revealed that *C10orf71* was mainly expressed in ventricular CMs (Supplemental Figure 13). Immunofluorescence analysis using mouse CMs revealed that mC10orf71 colocalized with markers of Z disc (Actn2) and myofibers (Actc1) (Figure 2E). As human CMs were not available, we validated this result by double-staining of hiPSC-CMs (Figure 2F). The specific and high expression of C10orf71 in the heart implies that it may play an essential role in the physiological and pathological processes of DCM.

### C10orf71 defect leads to abnormal heart morphogenesis.

**Figure 3:**
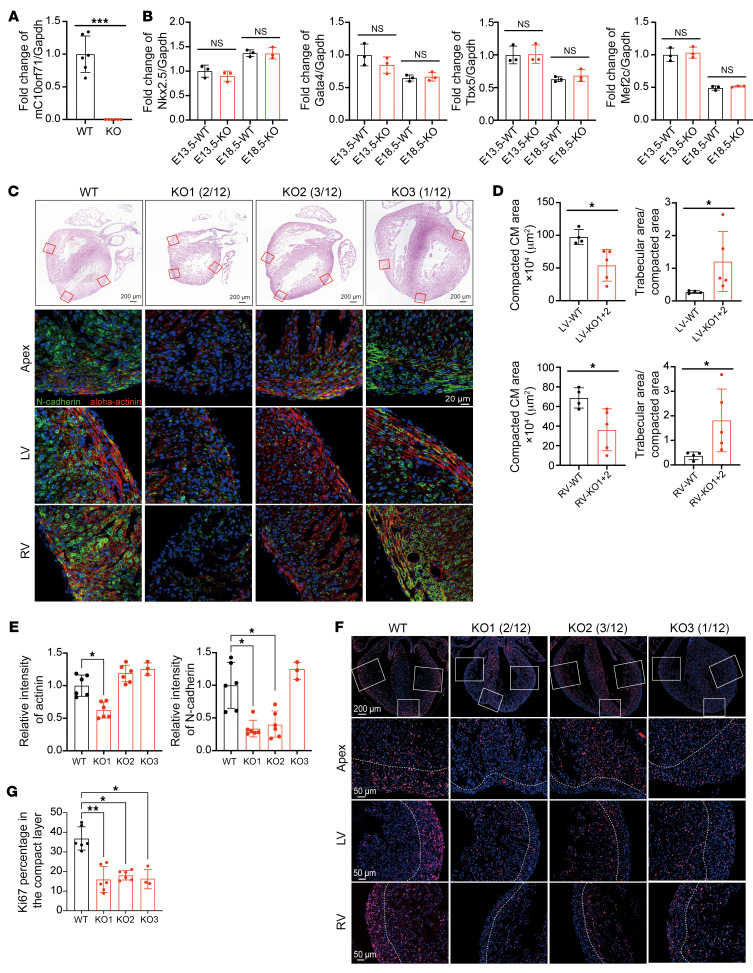
Phenotypes of embryonic heart in m*C10orf71*^–/–^ mice. (**A**) Relative mRNA levels of m*C10orf71* in E18.5 hearts (*n* = 6 per group). ***P < 0.001 in *t* test. (**B**) Relative mRNA levels of transcription factors in E13.5 and E18.5 hearts (*n* = 6 per group). NS, no significance in *t* test. (**C**) Representative H&E images for embryonic hearts (first line) and immunofluorescence staining of N-cadherin (green), alpha-actinin (red), and DAPI (blue) in heart apex, left ventricle (LV) and right ventricle (RV) (line 2–4). Scale bar: 200 μm (first line), 20 μm (lines 2–4). (**D**) Compaction degree of left and right ventricle shown in panel C (*n* = 4 for WT and *n* = 5 for m*C10orf71^–/–^*). *P < 0.05 in Mann-Whitney test. (**E**) The relative signal intensity of α-actinin and N-cadherin shown in panel C. *P < 0.05 in Kruskal-Wallis combined with Dunn’s multiple comparisons test. (**F**) Positive signal of Ki67 in embryonic hearts. The compact layer refers to the area between the dashed line and the epicardium as defined by higher cell density. Scale bar: 200 μm (first line), 50 μm (lines 2–4). (**G**) Statistical results of panel **F**: *P < 0.05, **P < 0.01 in Kruskal-Wallis combined with Dunn’s multiple comparisons test. Each dot in panel **A**, **B** and **D** represents 1 biological repeat. Each dot in panel **E** and **G** represents 1 region of a heart, and each mouse has 3 dots. Data is represented as mean ± SD.

To elucidate the function of C10orf71, we generated a m*C10orf71*-KO mouse model using the CRISPR/Cas9 method, which simulated the functional deletion of the *C10orf71* gene caused by LOF variants (Supplemental Figure 14). We mated heterozygous offspring to obtain m*C10orf71*^^–/–^^ mice. qPCR and Western blotting using heart tissue from m*C10orf71*^^–/–^^ mice and WT littermate controls confirmed the KO efficiency (Figure 3A and Supplemental Figure 15). Since m*C10orf71* expression increased with heart development (Figure 2C), we first evaluated the impact of mC10orf71 deficiency on embryo viability and heart development. We collected embryos at E13.5 and E18.5, and genotypes in E18.5 embryos were present at the expected Mendelian ratios, indicating no embryonic lethality. Expression of transcription factors important for cardiac development (*Nkx2-5*, *Gata4/6*, *Mef2C*, and *Tbx5*) did not differ in KO hearts compared with WT hearts at either time point (Figure 3B), suggesting that the overall cardiac differentiation program was unaffected. However, histological analysis found some cardiac morphogenesis defects in m*C10orf71*^^−/−^^ embryos (Figure 3C). Although half of the m*C10orf71*^^−/−^^ hearts looked similar to the WT, others showed variable phenotypes such as smaller size and noncompaction (2 of 12, KO1), normal size and noncompaction (3 of 12, KO2), and larger size and hypertrophy (1 of 12, KO3). Specifically, hearts with a ventricular noncompaction phenotype had reduced ventricular wall thickness and increased trabecular area compared with controls (Figure 3D). α-actinin and N-cadherin expression patterns correlate with progressive cardiac tissue development (13). We showed that the level of α-actinin in the small and noncompaction heart (KO1) was lower than that of WT (Figure 3, C and E). N-cadherin mediates strong cell-cell adhesion in the heart and is dispersedly distributed both on the cell surface and in the cytoplasm of myocardial cells at E18.5. It gradually becomes concentrated at the end-to-end intercalated discs from birth through maturity (14). The localization of N-cadherin in WT heart was as expected. However, the amount of N-cadherin in the KO heart with noncompaction (KO1 and KO2) was decreased compared with the WT heart (Figure 3, C and E). These results indicate abnormalities in the development and organization of myocardial cells in m*C10orf71*^^−/−^^ mice.

Because ventricular noncompaction has been shown to arise from defects in CM proliferation (15), we assessed proliferation markers in WT and KO E18.5 hearts. We observed an overall low Ki67 signal in smaller and noncompaction KO hearts (KO1) (Figure 3F). For the heart with normal size but noncompaction (KO2), the overall Ki67 signal was not significantly lower but its distribution was significantly different from WT hearts. The embryonic ventricular wall can be divided into different layers, including the compact layer and the trabecular layer (16). We found dramatically reduced proliferation of the CMs in the compact layer in KO mice (Figure 3, F and G), which was crucial for the development of postnatal ventricular walls that are largely composed of compact myocardium. These observed abnormalities in heart morphogenesis predicted poor consequences for m*C10orf71*^^−/−^^ mice after birth when the cardiac workload dramatically increases. As expected, of the 326 live offspring at weaning, m*C10orf71*^^+/−^^ and m*C10orf71*^^−/−^^ mice were less frequent (38.3% and 13.5%, respectively) than expected (*P* = 2.64 × 10^^–9^^, Supplemental Table 2), suggesting that m*C10orf71*-deficient mice were dying after birth. We collected the hearts of mice at 1 and 3 weeks after birth for histological analysis and did not find any KO hearts with noncompaction, suggesting that KO mice with noncompaction hearts (KO1 and KO2) were lethal. Hearts with larger size and hypertrophy (KO3) can be found in 2 of 13 mice at 1 week and 1of 10 mice at 3 weeks (Supplemental Figure 16). Taken together, these results clearly establish that m*C10orf71* is an essential gene for heart morphogenesis, although its loss does not interfere with the overall patterning of heart development.

### C10orf71 defect impairs cardiac function.

**Figure 4:**
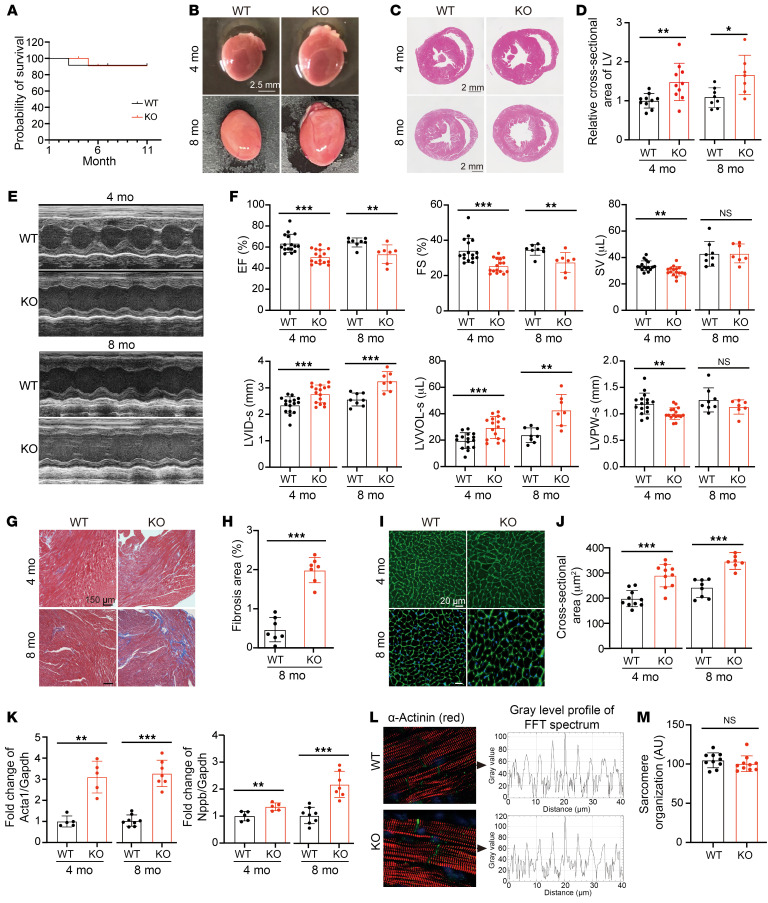
Phenotypes of heart in adult m*C10orf71*^–/–^ mice. (**A**) Survival rate of WT and m*C10orf71*^–/–^ (*n* = 10 per group) mice. (**B**) A photograph of hearts from 4 and 8-month-old mice (4 mo; 8 mo). Scale bar: 2.5 mm.(**C**) Histological analysis of hearts by H&E staining. Scale bar: 2 mm. (**D**) Quantification of cross-sectional area of left ventricle shown in panel **C** (*n* = 10 per group at 4 months; *n* = 7–8 at 8 months). *P < 0.05, **P < 0.01 in *t* test. (**E**) Representative echocardiographic images. (**F**) Echocardiographic parameters (*n* = 16 per group at 4 months; *n* = 7–8 at 8 months). EF, ejection fraction; FS, fraction shortening; SV, stroke volume; LVID-s, internal dimension of left ventricle at end systole; LVVOL-s, left ventricular volume at end-systole; LVPW-s, posterior wall thickness of LV at end-systole. **P < 0.01, ***P < 0.001 in *t* test. (**G**) Masson staining of hearts. Scale bar: 150 μm. (**H**) Quantification of fibrosis area at 8 months shown in panel **G**. ***P < 0.001 in *t* test. (**I**) WGA staining of hearts. (**J**) Quantification of cross-sectional area of CMs shown in panel **I** (*n* = 10 per group at 4 months; *n* = 7–8 at 8 months, *n* = 250–350 cells per mouse). ***P < 0.001 in *t* test. Scale bar: 20 μm. (**K**) Relative mRNA levels of *Acta1* and *Nppb* in hearts (*n* = 5 per group at 4 months; *n* = 7–8 at 8 months). **P < 0.01 in Mann-Whitney test. ***P < 0.001 in *t* test. (**L** and **M**) TT power analyses of the sarcomere organization were carried out with TTorg plugin in ImageJ. TTorg workflow of the sample images: magnification of an original image, 2D fast Fourier transformation (FFT) spectrum of the image, grey level profile of the FFT spectrum and analysis results. AU, arbitrary units. Each dot represents 1 biological repeat. Data is represented as mean ± SD.

Starting from 3 weeks of age, m*C10orf71*^^−/−^^ mice showed no increased mortality through 11 months of age compared with WT mice (Figure 4A). The KO hearts were morphologically normal but larger than hearts of WT mice at 4 and 8 months (Figure 4B). Mean KO heart weight was significantly decreased compared with WT at 4 months, but no difference was observed at 8 months (Supplemental Figure 17). H&E staining showed increased left ventricular cross–sectional area in KO mice compared with WT mice at 4 and 8 months (Figure 4, C and D).

We then used cardiac ultrasound to detect the cardiac function. Echocardiograms (Figure 4E) showed that the heart rate of KO mice was not significantly different from their WT littermates at 4 months of age but was lower at 8 months (Supplemental Figure 18A). KO mice exhibited lower EF and fraction shortening (FS) as well as stroke volume (SV) compared with the controls (Figure 4F) at 2 time points. Additionally, KO mice had increased LVID and left ventricular volume (LVVOL) as well as decreased left ventricular posterior wall thickness (LVPW) at end systole (Figure 4F), consistent with histology results and suggesting dilation of the KO heart. LVID and LVVOL also showed a trend to being higher at end diastole, but the difference was not as great as that at end systole (Supplemental Figure 18B), suggesting that the problem of contraction is more serious than that of relaxation. In addition, although there was no significant change in cardiac function in 8-month-old KO mice compared with 4-month-old KO mice, the degree of left ventricular dilation at 8 months increased. To further determine whether the DCM phenotype progressed with subsequent aging, we next tested the cardiac function of 53–63 week-old mice (Supplemental Figure 19). Supplemental Table 3 showed that the mean EF of the older KO mice was 39%, while it was 53% at 8 months, and the mean FS of older KO mice was 19%, while it was 27% at 8 months. The degree of left ventricular dilation in older KO mice further worsened. The LVIDs and LVVOLs were 3.58 mm and 54.02 μL in mice at 53–63 weeks, respectively, while they were 3.24 mm and 42.72 μL at 8 months. These data indicated the progression of cardiomyopathy with time.

Both 4-month-old KO mice and WT mice showed no substantial cardiac fibrosis. However, by 8 months of age, the cardiac fibrosis in KO mice significantly increased compared with the WT mice (Figure 4, G and H). Wheat germ agglutinin staining showed that the cross-sectional area of CMs was greater in both 4-month-old KO mice and 8-month-old KO mice (Figure 4, I and J). Early hallmarks of heart failure, including skeletal muscle α-actin (Acta1) and brain natriuretic peptide (Nppb) were upregulated in KO hearts (Figure 4K). However, confocal microscopy of α-actinin stained cells showed that organization of contractile apparatus in KO-CMs were not affected (Figure 4, L and M). This was confirmed by tissue transmission electron microscopy (Supplemental Figure 20).

Since the patients in this study were all heterozygous, we also evaluated the cardiac phenotype of heterozygous mice. We collected 7–8-month-old heterozygous mice as well as their WT and KO littermates. There was no significant difference in either the heart mass–to–tibial length or the heart mass–to–body weight among 3 groups (Supplemental Figure 21). Echocardiography showed the significant cardiac dysfunction in heterozygous mice compared with WT mice, and there was no significant difference between heterozygous and homozygous KO mice (Supplemental Figure 22). The above results were all from male mice. Due to the fact that patients involved both males and females, we also evaluated the cardiac function of 4–8-month-old female mice with different genotypes. Similarly, the obvious cardiac functional defects observed in male mice with m*C10orf71* deficiency also appeared in female mice (Supplemental Figure 23), indicating that the function of mC10orf71 was not affected by gender.

Collectively, these data support that the deletion of m*C10orf71* impairs cardiac function and causes a DCM-like phenotype in adult mice.

### C10orf71 defect affects the expression and splicing of contractile genes.

**Figure 5:**
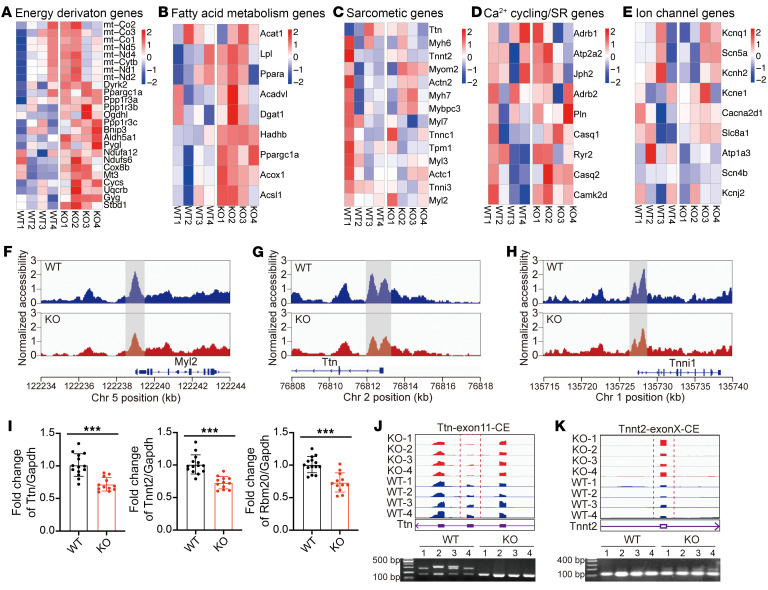
*C10orf71* deletion affects the expression and splicing of contractile genes. (**A**) Heatmap of expression values for genes related to energy generation. (**B**–**E**) Heatmap of expression values for genes related to fatty acid metabolism (**B**), sarcomere (**C**), Ca^2+^ cycling/SR (**D**), and ion channels (**E**). (**F**–**H**) IGV view showing normalized accessibility of promoters of target genes. (**I**) qPCR validation of expression changes of *Ttn*, *Tnnt2*, and *Rbm20* shown in **K** (*n* = 12–14). ***P < 0.001 in *t* test. (**J** and **K**) IGV view showing reads mapping to exons of *Ttn* and *Tnnt2* (**J**) and RT-PCR validation of alternative splicing (**K**). Each dot represents 1 biological repeat. Data represent means ± SD.

To explore the specific functional and cellular processes that were affected by the m*C10orf71* deletion, we compared total RNA expression in samples from hearts from E18.5. Data of E18.5 mice showed that there are 344 downregulated genes and 327 upregulated genes. Gene Ontology (GO) enrichment analysis showed that downregulated genes were not significantly enriched in any biological process. The 327 genes upregulated in KO mice were enriched for energy generation, electron transport chain and ATP synthesis, and many of them are encoded by mitochondrial DNA (Table 2 and Figure 5A). These changes were likely to be a compensatory response to the impaired morphogenesis and dysfunction of the KO hearts. We selected a series of genes that are crucial for heart maturation and normal function and compared their expression level between WT and KO hearts. There were no significant differences in the expression of Ca^^2+^^ cycling/SR, sarcomeric, or ion channel genes, except for a trend of increased expression of fatty acid metabolism–related genes in KO hearts (Figure 5, B–E).

The perinatal period is a critical window for CMs because they must change rapidly to accommodate the switch from fetal to neonatal circulation. CM perinatal transition is a highly coordinated process involving a well-orchestrated transcription regulation program (17). To assess transcriptional regulation program in WT and KO hearts, we performed ATAC-Seq of hearts at E18.5. In KO hearts, genes exhibiting a higher open chromatin in promoter regions were enriched for small GTPase mediated signal transduction (Table 3), while genes exhibiting a lower open chromatin positioning were enriched for the terms related to heart contraction (Table 3 and Supplemental Table 4). Chromatin accessibility of promoter regions decreased for *Myl2*, *Ttn*, and *Tnni1* (Figure 5, F–H), thereby demonstrating that the expression potential of these genes was decreased in E18.5.

Then, we performed RNA-Seq on hearts of adult WT and KO mice. 1,253 genes were differentially expressed, of which 640 were upregulated and 613 downregulated in KO mice. Consistent with the result of ATAC-Seq at E18.5, GO analysis of downregulated genes identified 2 categories of biological processes: mRNA processing/splicing and muscle cell differentiation/contraction (Table 4). Known DCM causal genes were among the downregulated genes, including *Ttn*, *Tnnt2*, *Pln*, *Nexn*, *Rbm20*, and *Rbm24* (Supplemental Figure 24, A and B), also validated by qPCR with larger sample size (Figure 5I and Supplemental Figure 24C). Expressional differences in mRNA splicing–related genes led us to assess alternative splicing (AS) of genes in KO hearts using rMATs software. We identified 510 AS events in a total of 437 genes (Supplemental Figure 25) with cassette exon splicing accounting for 42.7%. GO analysis of genes with AS suggested that the most significantly enriched biological processes were muscle differentiation and contraction (Table 5 and Supplemental Table 5). Genes involved in CM contractile function, such as *Ttn* and *Tnnt2* were confirmed by RT-PCR (Figure 5, J and K). When we compared the differentially expressed genes with genes with differential AS, we found 6 contraction-related genes in both groups: *Ttn*, *Tnnt2*, *Myl1*, *Pln*, *Atp1a2*, and *Trdn*. Although the differentially expressed genes (DEGs) at E18.5 were not enriched in terms of RNA splicing, the changes in adult mice drove us to examine whether there were any changes in contractile gene splicing at that time. Results showed that there were already splicing changes in contractile genes at E18.5 (Supplemental Table 6). Consistent with the results in adult mice, Tnnt2 was the gene with the most significant changes at E18.5 (Supplemental Figure 26). The main biological process of upregulated gene expression in adult KO mice was extracellular matrix organization (Table 4 and Supplemental Figure 27), a consequence of pathological remodeling of the heart, and was consistent with the heart phenotype. Similar to the results at E18.5, Ca^^2+^^ cycling/SR, ion channel genes, and fatty acid metabolism-related genes were not significantly different in KO hearts (Supplemental Figure 28). Collectively, our data strongly support that *C10orf71* KO leads to changes in gene expression and splicing of contraction related genes, negatively impacting the contractile function of CMs.

### C10orf71-defective CMs exhibit impaired contractile function.

**Figure 6:**
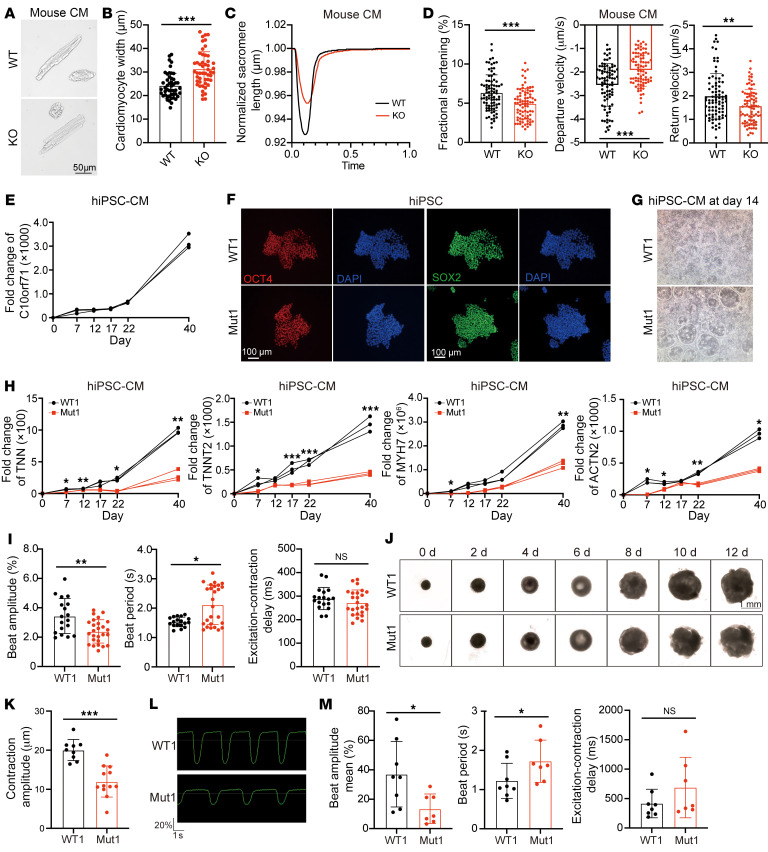
*C10orf71*-defective CMs exhibit impaired contractile function. (**A**) Representative images for cells used in IonOptix measurements. (**B**) Quantification of CM width shown in panel **A** (*n* = 48–50). ***P < 0.001 in *t* test. (**C**) Representative traces of sarcomere shortening in paced ventricular myocytes isolated from WT and KO mice. (**D**) Sarcomere shortening (expressed as the percentage of resting sarcomere length, SL), departure velocity, and return velocity in WT and KO CMs. *P < 0.05, **P < 0.01 in *t* test. (**E**) Relative mRNA levels of *C10orf71* during WT1 hiPSC-CMs differentiation (*n* = 3 independent differentiations). (**F**) Representative images showing immunofluorescence staining of pluripotent markers (OCT4 and SOX2) in WT1 and Mut1 iPSCs. DAPI, nuclei stain, blue. Scale bar: 100 μm. (**G**) Representative images for WT1 and Mut1 monolayer hiPSC-CMs sheets. (**H**) Relative mRNA levels of *TTN, TNNT2, MYH7,* and *ACTN2* during WT1 and Mut1 hiPSC-CMs differentiation (*n* = 3 independent differentiations). *P < 0.05, **P < 0.01, ***P < 0.001 in 2-way ANOVA followed by Šidák’s post hoc test. (**I**) MEA parameters for WT1 and Mut1 hiPSC-CMs differentiated for 40 days, including beat amplitude, beat period, and excitation-contraction delay (*n* = 18 for WT1 and *n* = 26 for Mut1). **P < 0.01, *t* test. *P < 0.05 in Mann-Whitney test. (**J**) Morphological changes of iPSC-HO at differentiation stages. Scale bar: 1 mm. (**K**) Contractility based on dynamic morphological information (*n* = 9 for WT1 and *n* = 12 for Mut1). ***P < 0.001 in *t* test. (**L**) FP waveforms of WT1 and Mut1 iPSC-HO. (**M**) MEA parameters for WT1 and Mut1 iPSC-HO, including beat amplitude, beat period, and excitation-contraction delay (*n* = 8 for WT1 and *n* = 7 for Mut1). *P < 0.05, *t* test. Each dot represents 1 biological repeat. Data represent mean ± SD.

To directly demonstrate that C10orf71 deficiency results in contractile dysfunction of CMs, we performed IonOptix measurements in primary isolated CMs from WT and m*C10orf71* KO hearts. Although KO CMs showed an increased width (Figure 6, A and B) and slightly decreased sarcomere length (Supplemental Figure 29A), the arrangement of sarcomere did not show obvious disorder, consistent with previous results in vivo (Figure 4, L and M). However, the KO CMs had a significantly reduced shortening and reduced contraction and relaxation speeds compared with WT CMs (Figure 6, C and D). There was no significant difference in time-to-peak, time-to-50% peak, or time-to-50% relaxation (Supplemental Figure 29B).

To simulate the specific defects caused by *C10orf71* variants in human CMs, we next detected the contractile function of homozygous c.173dupA and c.172_182del mutant hiPSC-CMs. *C10orf71* was minimally expressed in undifferentiated WT1 and WT2 hiPSCs and continuously increased during the differentiation process (Figure 6E and Supplemental Figure 30A). After 11–12 days of differentiation, WT hiPSC-CMs began to beat. Although *C10orf71*-mutant hiPSCs demonstrated normal pluripotency based on expression of pluripotent markers (*OCT4*, *SOX2*, *NANOG*, and *SSEA4*) (Figures 6F and Supplemental Figure 31), they began to beat 1–2 days later than WT hiPSC-CMs. A significant defect in differentiation efficiency can be found in mutant cells at day 14 (Figure 6G and Supplemental Figure 30B). *C10orf71*-mutant hiPSCs were able to differentiate into monolayer CM sheets but had a dramatically weaker spontaneous beating activity, which was distinct from the strong and synchronized spontaneous-beating monolayer of control CM sheets at day 40 (Supplemental Videos 1 and 2). The dynamic expression of CM marker genes also differed between the 2 groups. Expression of *TTN*, *TNNT2*, *MYH7,* and *ACTN2* were significantly reduced in the mutant compared with the control cells during the differentiation process (Figure 6H and Supplemental Figure 30C). Further functional characterization revealed a reduced beat amplitude and an increased beat period of the mutant cells (Figure 6I and Supplemental Figure 30D). However, the excitation-contraction delay was unchanged in *C10orf71*-mutant hiPSC-CMs (Figure 6I and Supplemental Figure 30D), suggesting the electrophysiological activity was not affected by the *C10orf71* mutation. To further clarify the changes in hiPSC-CMs carrying heterozygous *C10orf71* mutations, we constructed another heterozygous mutant hiPSC line (Mut3: c.164_173del) using WT1 hiPSC line (Supplemental Figure 32A). It can be found that the defects of heterozygous mutant hiPSC-CMs were similar to those found in homozygous mutant cells, including decreased expression of CM marker genes and beat amplitude (Supplemental Figure 32, B and C).

We further assessed the phenotype in hiPSC-derived heart organoids (hiPSC-HOs). As the differentiation progressed, we observed that the morphology and size of the hiPSC-HOs changed, but there was no significant difference between WT1 and Mut1 (homozygous c.173dupA) groups (Figure 6J). We selected chamber-formed hiPSC-HOs that account for the largest proportion of the morphologies of the developed hiPSC-HOs at day 6 for further analysis. The diameter of WT1 hiPSC-HOs was 1257.8 ± 167.9 μm and 1294.4 ± 117.9 μm for the Mut1 hiPSC-HOs at day 12. Immunofluorescence showed that both WT1 and Mut1 hiPSC-HOs were mainly composed of myocardial cells and a small number of endothelial cells (PECAM) (Supplemental Figure 33). The video on the 12th day showed that the beating of Mut1 hiPSC-HOs was weak (Supplemental Videos 3 and 4). We analyzed the contractility based on dynamic morphological information. Contraction distance of WT1 hiPSC-HOs was significantly larger than that in Mut1 group (Figure 6K). MEA assay also showed a reduced beat amplitude and an increased beat period in Mut1 hiPSC-HOs compared with WT1 (Figure 6, L and M). Similarly, contraction distance of Mut3 (heterozygous c.164_173del) hiPSC-HOs was also decreased compared with WT1 (Supplemental Figure 34).

In summary, these data from hiPSC-CMs and hiPSC-HOs showed that the *C10orf71* defect led to the reduction in hiPSCs differentiation efficiency and reduced contractile function of differentiated CMs. As *C10orf71* does not affect the sarcomere structure of myocardial cells, we speculated that the effects on contraction were mainly due to altered mechanical activities, such as force generation and conduction.

### Candidate myosin activator rescues cardiac contractile dysfunction caused by C10orf71 deficiency.

**Figure 7:**
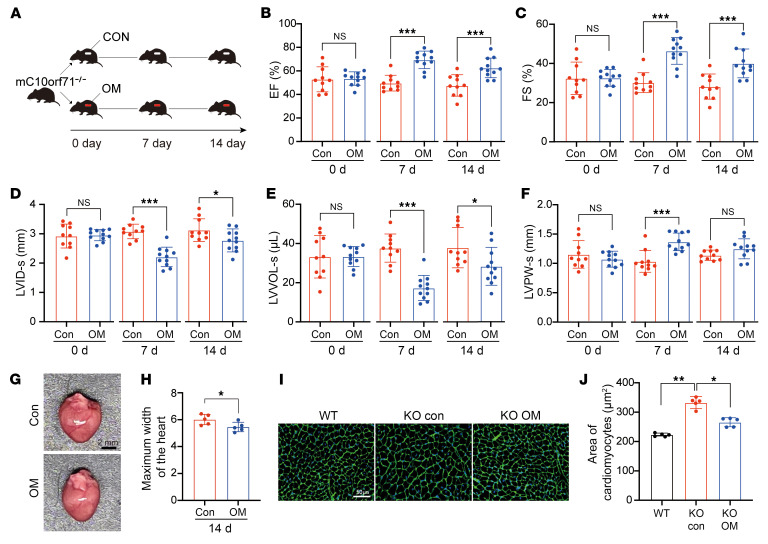
OM rescues cardiac contractile dysfunction caused by *C10orf71* deficiency. (**A**) Flow chart of OM treatment and echocardiographic testing. (**B**–**F**) Echocardiographic parameters (*n* = 5 males and 5–6 females per group at 0, 7, 14 days after OM treatment). EF, ejection fraction; FS, fraction shortening; SV, stroke volume; LVID-s, internal dimension of left ventricle at end-systole; LVVOL-s, left ventricular volume at end-systole; LVPW-s, posterior wall thickness of LV at end-systole. *P < 0.05, ***P < 0.001 in *t* test. (**G**) A photograph of hearts from OM treated and control mice. Scale bar: 2 mm. (**H**) Quantification of maximum width of the hearts shown in panel **G** (*n* = 5 males per group). *P < 0.05 in Mann-Whitney test. (**I**) WGA staining of hearts from OM treated and control mice. Scale bar: 50 μm. (**J**) Quantification of cross-sectional area of CMs shown in panel **I** (*n* = 5 males per group, *n* = 250–300 cells per mouse). *P < 0.05, **P < 0.01 in Kruskal-Wallis combined with Dunn’s multiple comparisons test. Each dot represents 1 biological repeat. Data represent mean ± SD.

We demonstrated that the cardiac phenotype caused by *C10orf71* defect was mainly due to dysfunction in the contractile function of the CMs. Therefore, drugs targeting sarcomere function may compensate for the *C10orf71* defect. Omecamtiv mecarbil (OM) was a lead candidate myosin activator, and we tested the effects of OM in m*C10orf71* KO mice including both males and females. 12-week-old KO mice were randomly divided into OM treatment and control group, and echocardiography was performed after 0, 7, and 14 days of OM treatment (Figure 7A). OM treatment for 7 days and 14 days significantly improved EF and FS (Figure 7, B and C), reduced LVID and LVVOL, and increased LVPW at end systole (Figure 7, D–F) compared with no treatment. The OM-treated mice had smaller hearts (Figure 7, G and H) and a reduced cross-sectional area of CMs compared with no-treatment mice at 14 days (Figure 7, I and J). We also tested the effect of OM on heterozygous mice, and results showed that OM could also rescue the phenotype of heterozygous mice (Supplemental Figure 35).

To explain the mechanisms by which OM helps normalize the phenotype, we carried out transcriptional analysis of KO heart tissues with and without OM treatment. Results showed that genes upregulated by OM treatment were enriched for the terms related to CM differentiation and contraction (Supplemental Figure 36A), which was exactly the terms downregulated in adult KO hearts. Many upregulated genes in these terms were overlapped with those decreased in adult KO hearts (Supplemental Figure 36B). There were also other contractile genes that did not decrease in adult KO hearts but significantly increased after OM treatment (Supplemental Figure 36C). Compared with contractile genes, OM treatment had less significant changes in genes related to fatty acid metabolism (Supplemental Figure 36D), Ca^^2+^^ cycling/SR (Supplemental Figure 36E), as well as ion channels (Supplemental Figure 36F). These results indicate that OM mainly improves the phenotype of DCM by enhancing CM contraction. The top 8 downregulated terms were shown in Supplemental Figure 37, 6 of which were related to protein catabolic process.

## Discussion

We identified *C10orf71* as a gene for DCM through human genetic studies and in vivo functional studies in mice and in vitro models derived from human cells. Genetic analysis identified rare monoallelic *C10orf71* LOF variants in patients with familial and sporadic DCM. Functional studies showed that *C10orf71* was expressed in the heart and played a key role in heart morphogenesis and cardiac contractile function. Notably, C10orf71 is an intrinsically disordered protein, which represents a different, unknown category of DCM genes.

Our findings have several major implications. We identify a category of causal gene for DCM, enhancing the understanding of DCM genetic etiology. We provide detailed studies for *C10orf71*, a gene with poorly defined function previously. Our results elucidate the pathogenic mechanism of *C10orf71* LOF and suggest that the myosin activator OM has therapeutic effects on cardiac dysfunction caused by the *C10orf71* LOF variants.

### C10orf71 is a DCM gene with distinctive characteristics.

Several lines of genetic evidence support the causal role of ultra-rare *C10orf71* LOF variants in DCM. First, variants cosegregated with DCM in a large family with dominant transmission; none of the patients described had variants in other known DCM genes. Second, variants were found in sporadic DCM patients but absent in their healthy parents. Third, the patients with DCM with *C10orf71* variants were collected by 3 research groups independently, and the patients were of different genetic ancestry. Finally, all LOF variants were absent in gnomAD and in over 1,000 control individuals without cardiomyopathies. The variants were also absent in the dbSNP (build 132). It is worth noting that the human phenotypes show some variability. Two young patients had LVNC, while the older patients did not. It has been reported that LVNC is a variable morphological phenotype within some DCM cases (18). However, because we do not have echocardiograms of the older patients during their youth, we cannot rule out the possibility that they had LVNC in their early years.

Unlike DCM causal genes encoding a wide variety of proteins with stable secondary or tertiary structure and functions in the CM sarcomere, cytoskeleton, and nucleus, *C10orf71* is a DCM gene encoding an intrinsically disordered protein. Although we did not have an appropriate method to prove C10orf71 to be an intrinsically disordered protein through direct experimental approaches such as X-ray crystallography, due to the lack of highly purified C10orf71 proteins, we demonstrated that C10orf71 was an intrinsically disordered protein based on various disordered prediction methods. Besides the structure prediction, C10orf71 was calculated to fit to multiple characteristics of intrinsically disordered protein, such as high hydrophilicity and instability. Intrinsically disordered proteins are widely involved in important processes such as signal transduction, DNA transcription, cell division, and protein aggregation. Given their wide range of biological functions, it is not surprising that they are involved in human diseases including neurodegeneration and cancer (19). In this study, we link an intrinsically disordered protein with cardiomyopathy.

### Our study provides functional characteristics for C10orf71.

The C10orf71 protein was identified in 2017, but the function remained largely unknown (20). In vitro experiments previously described increased *C10orf71* expression leading to induction of CM hypertrophy. In vivo experiments of the role of C10orf71 in the pathogenesis of cardiomyopathy have not been reported. In this study, we elucidated the role of *C10orf71* in heart morphogenesis and contraction. Maintenance of the contractile function required a normal contraction apparatus and ion channel–based electrophysiological activity, and *C10orf71* mainly affected the function of the contraction apparatus. *C10orf71* deficiency affected the expression and splicing of contraction-related genes in adult KO mice. Although the contractile gene expression was unaltered at E18.5, chromatin accessibility was decreased around the promoter regions of many contractile genes. Chromatin accessibility is defined as the degree to which DNA can be bound by factors (21). This ‘‘time lag’’ between chromatin accessibility and gene expression has been found in many previous studies. For example, Ranzoni et al. observed that promoters of GATA1-targeted genes were often open prior to any noticeable gene expression in HSCs/MPPs (22). Jingyi Wu et al. found that many promoters became accessible in human 2C embryos while transcription from these promoters did not occur until 8C stage (23). These findings collectively indicate that chromatin accessibility of the promoter region in former stage may represent the potential for later gene expression, and the establishment of the early chromatin landscape may be important for the timely subsequent transcription (24). Similarly, splicing changes can also be traced back to E18.5. It is well known that cardiac dilation has a marked affect on gene expression and splicing. Due to the lack of cardiac dilation at E18.5, the gene expression and splicing changes are affected greatly by the direct effects of C10orf71 deletion. However, C10orf71 is mainly located in the Z-disc and myofilaments, and we have no evidence indicating that it directly regulates gene transcription in the nucleus. Further study is still needed to clarify the detailed regulatory mechanism.

### C10orf71 may regulate the contractile function of CMs by binding to contractile proteins.

Discovering the pathogenic genes provides insight into the pathological mechanism of DCM. As C10orf71 lacks homology with other proteins, we cannot infer the molecular mechanism of C10orf71 defects through its homologous proteins. Unlike the classical protein structure-function paradigm, intrinsically disordered proteins introduce a new paradigm of “coupled binding and folding”, which means that intrinsically disordered proteins typically participate in various biological processes by regulating functions of their binding partners (25). Intrinsically disordered proteins may participate in one or many interactions (26), binding to different partners under different conditions. A previous study has reported several potential binding proteins, such as ACTN2 and VIM, in yeast 2-hybrid screens using 3 protein fragments of C10orf71 as bait (20). The binding partners of sarcomeric proteins may act as structural proteins or as regulatory or signaling proteins. Previous fluorescence recovery after photobleaching has revealed that the dynamics and mobility of C10orf71 are different from other sarcomeric proteins in mammalian muscle cells, implying that C10orf71 is more likely to be a regulatory or signaling protein regulating the function of contractile proteins, thereby affecting the contraction of CMs and the heart.

### A contractile protein activator is a potential therapeutic drug for DCM caused by variants in contractile-related genes.

Pharmacologic treatment options for patients with DCM are not gene-specific and focus on controlling symptoms without addressing the disease mechanism (27). It has been hypothesized that direct activation of the contractile proteins without systemic effects on other organs would be a more effective treatment approach (28). OM is the first-in-class cardiac myosin activator (myotrope) discovered by high-throughput screening in 2010 without the disadvantages of classic inotropic agents including increasing oxygen demand, heart rate, and intracellular calcium, which are linked to hypotension, arrhythmias, and mortality (29, 30). It has been proven to improve cardiac function and reduce cardiovascular death or heart failure events (31–34). We tested the effect of OM on m*C10orf71*^^–/–^^ mice and observed therapeutic effects. Therefore, contractile protein activators such as OM may be promising targeted drugs in the setting of cardiomyopathy caused by variants in contractile-related genes.

### Limitation.

Our study has several limitations. First, we focused on LOF variants and might have underestimated the contribution of *C10orf71* to DCM. Second, although we identified *C10orf71* as the causal gene in several unrelated families of different ethnic backgrounds, the sample size in this study was not large. Therefore, the proportion of patients with DCM explained by this gene cannot be precisely estimated, so studies in larger cohorts are warranted. Third, this study focused on DCM, and screening for *C10orf71* variants in other cardiomyopathy cohorts could inform other types of cardiomyopathies including LVNC. Fourth, we speculated that C10orf71 might interact with sarcomere proteins. However, further studies are needed to prove this and provide a deeper understanding of how the *C10orf71* variants cause DCM.

### Conclusion.

Collectively, human genetic as well as in vivo and in vitro functional data implicate *C10orf71* as a causal gene for DCM. Translation of this research to the clinic via genetic testing may identify those who are at high risk for developing cardiomyopathy and perhaps an effective treatment strategy.

## Methods

### Sex as a biological variable

Our study examined male and female animals, and similar findings are reported for both sexes.

### Human participants

#### Multigenerational family.

A multigenerational family from Puerto Rico was recruited by Columbia University Irving Medical Center for a genetic research study based on clinical diagnosis of familial DCM. Seven family members were enrolled for WES, including 5 affected members. As described previously (35), DNA was extracted from peripheral blood leukocytes using Puregene reagents (Gentra Systems Inc.). Genomic DNA was prepared with a customized reagent kit from Kapa Biosystems and captured using the NimbleGen SeqCap VCRome 2 exome capture reagent or xGen lockdown probes. Samples were sequenced on the Illumina HiSeq 2500 platform with v4 chemistry, generating 75 bp or 76 bp pair end reads. We achieved coverage of ≥ 15 × in ≥ 90% of targeted regions for all WES samples. Given the family history suggestive of dominant inheritance, shared heterozygous variants among affected family members were assessed. *C10orf71* genotyping was confirmed by Sanger sequencing.

#### Sporadic DCM cohorts.

Beijing Anzhen DCM cohort included 220 patients with sporadic DCM of Chinese ancestry, who were evaluated at Beijing Anzhen Hospital from the year 2014 to 2020. Included patients had a clinical diagnosis of isolated DCM under the age of 60 with no definite DCM family history. The Wuhan Tongji DCM cohort included 363 sporadic early onset DCM patients, 272 of whom have no deleterious variants and were used in the study (9).

DCM was defined as left ventricular or biventricular systolic dysfunction and dilatation not explained by abnormal loading conditions or by the coronary artery. Left ventricular dilatation was defined as z-scores of LVID > 2 SDs from normal according to nomograms corrected by body surface area and age. Systolic dysfunction was defined as an LVEF < 50%. DCM was diagnosed and identified by at least 2 experienced cardiologists. DCM family history was self reported by patients or first-degree relatives. Some of the patients under the age of 18 did not reach the standard for systolic dysfunction, and they were defined as enlarged heart rather than DCM.

### Sanger sequencing validation

Bidirectional Sanger sequencing of the candidate regions of *C10orf71* was performed using PCR primers in Supplemental Table 7. Variant annotation was based on reference sequence NM_001135196.1 and following nomenclature recommendations from the Human Genome Variation Society.

### Effect of variants on *C10orf71* expression level

A *C10orf71* expression vector was purchased from Sino Biological (HG27964-NF) and variants were introduced using a PCR-based strategy with a site-directed mutagenesis kit (TIANGEN, KM101) and specially designed mutagenic primers (Supplemental Table 7). Specificities of the mutagenic events were verified using Sanger sequencing. Plasmids were purified, and WT and mutant plasmids were transfected into HEK293T cells using Lipofectamine 3000 (Thermo Fisher Scientific, L3000-015) transfection reagent. After 48 hours, we harvested total protein for Western blotting.

### Protein extraction and immunoblotting

Total protein was extracted from tissues or cultured cells in lysis buffer containing protease/phosphatase inhibitors. Protein was quantified using Bradford colorimetric assay and separated on SDS-polyacrylamide electrophoresis gels before being transferred to nitrocellulose membranes. Membranes were blocked with 5% skim milk in TBST for 1 hour at room temperature. The membranes were incubated with the primary antibodies for Flag (1:2,000 dilution, Proteintech Group, 20543-1-AP), C10orf71 (1:1,000 dilution, Abcepta, R03673), and GAPDH (1:2,000 dilution, Zsbio, TA505454) at 4°C overnight. This was followed by incubation with infrared Dye 800-conjugated secondary antibodies (1:10,000 dilution, LI-COR Biosciences, 926-32210 and 926-32213) for 1 hour at room temperature. Images were quantified using the Odyssey infrared imaging system (LI-COR Biosciences).

### RNA extraction and quantitative real-time PCR

Total RNA was extracted using TRIzol according to the manufacturer’s protocol (Invitrogen, 15596018). RNA was reverse transcribed using a reverse transcription kit (Promega, A3500) and random hexamer primers. The RT reaction was set as follows: 25°C for 10 minutes; 42°C for 60 minutes; 95°C for 5 minutes. Real-time PCR was performed using SYBR Green II (Takara, RR820B) and described primers (Supplemental Table 7) with the BioRad iQ5 system at 95°C for 5 minutes followed 40 cycles of 95°C for 15 seconds and 60°C for 45 seconds.

### Single nucleus RNA-Seq

Frozen tissue (3–50 mg) was transferred to a prechilled sample dissociation tube. Nuclei were isolated for single nucleus RNA-Seq using the Chromium nuclei isolation kit. Nuclei suspensions were stained with 7-AAD (Miltenyi; 1:50) and sorted on a BD Fusion flow cytometer with a 100 μm nozzle. Nuclei were counted using a Countstar automated cell counter and then loaded into the 10× Chromium system using the single cell 3′ reagent kit v3 according to the manufacturer’s protocol. Following library construction, libraries were sequenced on an Illumina NovaSeq 6,000 sequencer with 150 bp paired-end reads. After quality control, raw reads were demultiplexed and mapped to the reference genome (GRCh38) using the 10× Genomics Cell Ranger pipeline with default parameters. All downstream analyses were performed using Cell Ranger and Seurat. For each gene and each cell barcode, unique molecule identifiers were counted to construct digital expression matrices. A gene with expression in more than 3 cells was considered as expressed, and each cell was required to have at least 200 expressed genes. Loupe Cell Browser was used to view results of Cell Ranger.

### Immunofluorescence

Frozen sections of tissues or cells were fixed using 4% polyformaldehyde and permeabilized with 0.3% Triton X-100 for 10 minutes followed by blocking solution (5% serum of secondary antibody species, i.e., goat or donkey) for 1 hour at room temperature. Primary antibodies, isotype controls, or blocking reagent were used to stain the samples. Isotype controls and blocking reagent were used as negative controls to validate specificity and eliminate background signal. Primary antibodies included rabbit polyclonal anti-Flag (1:200 dilution, Proteintech Group, 20543-1-AP), mouse monoclonal anti-ACTN2 (1:200 dilution, Sigma-Aldrich, A7811), mouse monoclonal anti-ACTC1 (1:50 dilution, Proteintech Group, 66125-1-lg) and rabbit polyclonal anti-N-cadherin (1:100 dilution, Servicebio, GB111273-100). Isotype controls included rabbit IgG isotype control (1:100 dilution, Thermo Fisher Scientific, 31235), mouse IgM isotype control (1:100 dilution, Thermo Fisher Scientific, 14-4752-81), and mouse IgG1 isotype control (1:100 dilution, Thermo Fisher, #14-4714-82). After incubating at 4°C overnight, the slices were washed with PBS 3 times and incubated with secondary antibodies in a dark environment for 1 hour. After washing with PBS, the slides were mounted with Vectashield mounting media containing DAPI. Images were captured using a Leica ST5 laser scanning confocal microscope.

### hiPSC-derived CMs culture

ZZUNEUi022-A and ZZUNEUi011-A are 2 hiPSC lines derived from healthy male urine cells and healthy female peripheral blood mononuclear cells, respectively. Both of them exhibit stable karyotype and ability to differentiate into 3 germ layers (ectoderm, mesoderm, and endoderm) (10, 11). hiPSCs were cultured with hiPSC media (Nuwacell Biotechnologies Co., Ltd.; RP01020) in 6-well plates precoated with matrigel (Corning, 354277). At 90% confluence, the media was sequentially exchanged to CardioEasy human CM differentiation complete medium I (CEHCDCM; CELLAPY, CA2004500) for 48 hours, CEHCDCM II (CELLAPY, CA2004500) for 48 hours, and then CEHCDCM III (CELLAPY, CA2004500) for 48 hours. In CardioEasy human myocardial purified complete medium (CEHMPCM; CELLAPY, CA2005100), non-CMs died off and highly purified CMs were harvested.

### Generation and validation of a *C10orf71*-deficient mouse model

A *C10orf71*-deficient mouse model was generated using a CRISPR/*Cas9* strategy. Murine *C10orf71* (m*C10orf71*) gRNA was inserted into the gRNA cloning vector. Both the m*C10orf71* gRNA and *Cas9* mRNA were generated using in vitro transcription. gRNA and *Cas9* mRNA were coinjected into fertilized eggs. The pups were genotyped using PCR followed by sequence analysis using primers (KO-F: 5′ GCAGCCATTTCGCCATATCC 3′; KO-R: 5′ GACTGAGAAAGTACAGGGTAGGG 3′). KO-positive male pups were mated with C57BL/6J female mice. Heterozygous KO mice were intercrossed to generate homozygotes. Animals were housed in a specific pathogen-free facility according to the guide for the care and use of laboratory animals (National Institutes of Health publication no. 86-23, revised 1996). The experiments were approved by the institutional animal care and use committee of Beijing Anzhen Hospital.

### Mouse embryo isolation and embryonic hearts sampling

For timed pregnancies, detection of vaginal plugs was measured as E0.5. Moreover, all embryos were staged according to Theiler’s criteria to ensure reproducibility of results. Genotyping was performed by PCR using genomic DNA from embryo tails. When sampling embryonic hearts, we treated the hearts with 10% KCl solution to ensure the cardiac arrest at diastolic phase. After fixing and embedding, we performed longitudinal continuous sectioning of the embryonic hearts. Only slices that include the left/right atrial appendages, left/right ventricles, and outflow tract were retained.

### Histology

Heart tissues were obtained and fixed in 4% paraformaldehyde solution for 48 hours and embedded in paraffin. 6-μm thick sections were deparaffinized and stained with H&E, Masson’s trichrome, or wheat germ agglutinin (WGA) according to the manufacturer’s protocols. The sections were imaged using light microscopy (H&E and trichrome) or fluorescence (WGA) and measurements made using Image J software.

### Echocardiography

Murine cardiac structure and function were monitored and measured with transthoracic 2-dimensional M-mode echocardiogram using a high-resolution micro-ultrasound system Vevo 2100 (VisualSonics) equipped with a 30-MHz transducer. Mice were anesthetized and then maintained with 1% isoflurane throughout the procedure. EF, FS, and other left ventricle (LV) parameters were measured. Measurements were performed by a single individual blinded to mouse genotype and averaged over 5 cardiac cycles.

### Transmission electron microscopy

Hearts were harvested and cut into 1-mm^^3^^ sections. The sections were subsequently fixed in 2.5% glutaraldehyde overnight, immersed in 1% osmium tetroxide in 0.1-M cacodylate buffer for 1 hour and incubated with 2% aqueous uranyl acetate for 2 hours. After dehydration with a graded series of ethanol solution, the samples were embedded and sliced into small grids. Grids were observed using a transmission electron microscopy (HT7800, HITACHI).

### RNA-Seq for heart and data processing

For embryonic mice, hearts were isolated from E18.5 embryos generated by heterozygous parents and stored in Trizol reagent prior to genotyping. 3 hearts per genotype were pooled together and total RNA was extracted. RNA quality was evaluated via the Lab-Chip technique (Agilent Bioanalyzer). The libraries were constructed using NEBNext UltraTM RNA Library Prep Kit for Illumina and then sequenced on Illumina HiSeq 2,000 platform. Relative expression (fragments per kilobase per million mapped reads, FPKM) was calculated using uniquely mapped pairs with Cufflinks software (Version 2.2.1). Down regulated DEGs from KO embryonic heart met the following criteria: (a) average FPKM in WT group > 2; (b) *P* value < 0.1; (c) fold change (mutant/WT) < 0.8. Upregulated DEGs met the following criteria: (a) average FPKM in KO group > 2; (b) *P* value < 0.1; (c) fold change (mutant/WT) > 1.2.

For adult mice, we extracted total RNA from hearts using Trizol. The process of library construction and sequencing was the same as described above. Down regulated DEGs from KO adult heart satisfied the following criteria: (a) average FPKM in WT group > 2; (b) *P* value < 0.05; (c) fold change (mutant/WT) < 0.9. Upregulated DEGs from KO adult hearts, satisfied the following criteria: (a) average FPKM in KO group > 2; (b) *P* value < 0.05; (c) fold change (mutant/WT) > 1.1. GO enrichment analysis was performed using Novomagic.

### Assay for transposase-accessible chromatin sequencing (ATAC-Seq)

Heart nuclear suspensions were obtained and resuspended in the Tn5 transposase reaction mix and TD buffer (Illumina) for 30 minutes at 37°C. Equimolar adapter1 and adapter2 were added after transposition, and PCR was performed to amplify the library. Libraries were purified with the AMPure beads and quality was assessed with Qubit. Clustering of the index-coded samples was performed on a cBot Cluster Generation System using TruSeq PE Cluster Kit v3-cBot-HS (Illumina) according to the manufacturer’s instructions. The library preparations were sequenced on Illumina Novaseq platform at Tianjin Novogene Bioinformatic Technology Co. Ltd., and 150 bp paired-end reads were generated. Reads containing adapter or poly-N sequences, or reads of low-quality, were removed and the remaining reads were aligned to the reference genome (GRCh38) using BWA (v0.7.12). Uniquely mapped (MAPQ ≥ 13) and deduplicated reads were used for further analysis. All peak calling was performed with MACS2 (version 2.1.0). Peaks of different groups were merged using ‘bedtools merge’ and mean RPM of each group was calculated. Peak-related genes were confirmed by ChIPseeker and GO enrichment analysis was performed.

### Adult mouse CM isolation and culture

Adult ventricular CMs were freshly isolated using a Langendorff perfusion apparatus (ADInstruments). Mice were euthanized under anesthesia, and hearts were separated and mounted on a Langendorff device. The hearts were retrogradely perfused for 5 minutes with oxygenated (100% O_2_) normal Tyrode solution (137.0 mM NaCl, 1.2 mM NaH_2_PO_4_, 5.0 mM KCl, 1.2 mM MgCl_2_, 10.0 mM HEPES, 10 mM glucose, 1.8 mM CaCl_2_, pH 7.4 [ all from Sigma-Aldrich]). Then, perfusate was switched to Ca^^2+^^-free Tyrode solution for 5 minutes, followed by perfusion for 25 minutes with the same solution plus 30 μM CaCl_2_ and 0.6 g/mL of type II collagenase (Worthington Biochemical). Next, hearts were dissociated gently in Ca^^2+^^-free Tyrode solution with 1% BSA to separate CMs. After filtration through a 100 μm strainer, cells were centrifuged at 400 rpm for 30 seconds and resuspended with extracellular Ca^^2+^^ added back to 1.8 mM. Calcium-tolerant and rod-shaped cells showing clear cross striations were used for sarcomere shortening measurements.

### Sarcomere shortening measurement

Isolated CM sarcomere shortening was recorded by Cell Dimensioning Systems (IonOptix) in the absence of fluorescent dye. Cells were placed in the cell chamber, stimulated at 1 hertz for 4 ms, and superfused at room temperature.

### hiPSC-HOs culture

The HOs were derived from the hiPSCs lines described above (ZZUNEUi022-A and ZZUNEUi011-A) using a kit for differentiation of cardiac organoids (MEGAROBO TECHNOLOGIES, MG-PSC-Co-24). Briefly, the iPSCs were induced to form embryonic bodies. Then, a defined method based on small molecule compounds optimized for CM differentiation was used to generate hiPSC-HOs. The hiPSC-HOs began to beating from 9-to-13 days after differentiation.

### Microelectrode array recordings

hiPSC-CMs were plated on 24-well microelectrode array (MEA) plates (Axion Biosystems Inc). hiPSC-HOs were placed in the center of the well with the electrodes. Contraction of hiPSC-CMs and hiPSC-HOs were measured using a Maestro Edge device (Axion BioSystems Inc.) according to the manufacturer’s protocol. Cardiac Analysis Tool, AxIS Navigator, AxIS data export tool, and Origin were used to analyze the data.

### Murine drug treatment

OM (M2004, Abmole Bioscience, 0.25 mg/kg/h) was resuspended in 20% DMSO (Sigma-Aldrich), 30% PEG300 (MedChemExpress), 1% Tween-80 (MedChemExpress) and delivered continuously by subcutaneously implanted mini-osmotic pumps (ALZET model 2002, 200 μl total volume). Treatment was initiated at 12 weeks of age for a total of 14 days. The minipumps were filled and prepared for implantation following the manufacturer’s instructions, with a designated pumping rate set at 0.5 μL per hour.

### Statistics

Outliers were identified using ROUT in Prism. For data with sample size greater than 6/group, the Shapiro-Wilk test was used to evaluate the data distribution normality. For data that passed the normality test, a 2-tailed *t* test combined with Levene’s test or 1-way ANOVA combined with Tukey’s post hoc test was used to compare means between groups. When sample size was small (≤ 6/group), nonparametric tests were used, including the Mann-Whitney and the Kruskal-Wallis, followed with Dunn’s post hoc test. Comparison of time-response curves between 2 groups was made using 2-way ANOVA followed by Šidáks post hoc test. Statistical analysis was performed with Prism version 8.0. A *P* value less than 0.05 was considered significant.

### Study approval

The study was conducted in agreement with the principles outlined in the Declaration of Helsinki. Written informed consent was obtained from all patients or their legal family members according to the research protocols approved by the ethical review board of Beijing Anzhen Hospital and Wuhan Tongji Hospital.

### Data availability

Values for all data points in graphs are reported in the Supporting Data Values file. Online databases used in this study include AlphaFoldDB (https://alphafold.ebi.ac.uk/), ProtScale (https://web.expasy.org/protscale/), ProtParam database (https://web.expasy.org/protparam/), GTEx (V8, https://www.gtexportal.org/), and Novomagic (https://magic.novogene.com). Next-generation sequencing data have been deposited in GEO (accession no. GSE264733). Any other data can be requested from the corresponding author.

## Author contributions

Yang Li and Yulin Li designed the study. Yang Li, Yujie Li, CW, ELG, PR, ZV, DWW, J Du, and WKC collected genetic samples and analyzed genetic data. Yang Li, KM, ZD, SG, JZ, SH, JY, GF, X Li, and J Dong conducted functional experiments. Yang Li, KM, ZD, and X Liu performed statistical analysis. Yang Li, CW, WKC, and Yulin Li wrote the manuscript.

## Supplementary Material

Supplemental data

Unedited blot and gel images

Supplemental video 1

Supplemental video 2

Supplemental video 3

Supplemental video 4

Supporting data values

## Figures and Tables

**Table 5:**
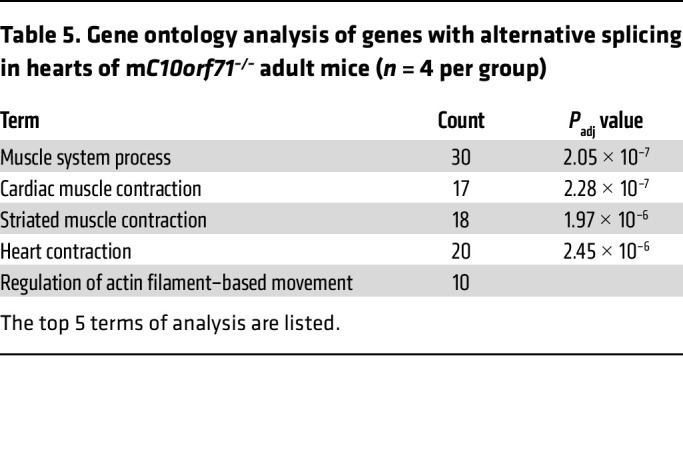
Gene ontology analysis of genes with alternative splicing in hearts of m*C10orf71^–/–^* adult mice (*n* = 4 per group)

**Table 4:**
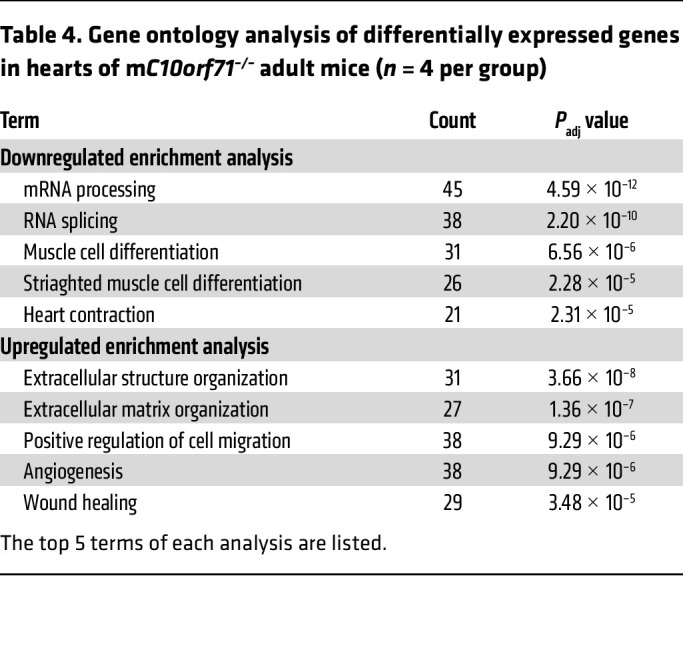
Gene ontology analysis of differentially expressed genes in hearts of m*C10orf71^–/–^* adult mice (*n* = 4 per group)

**Table 3:**
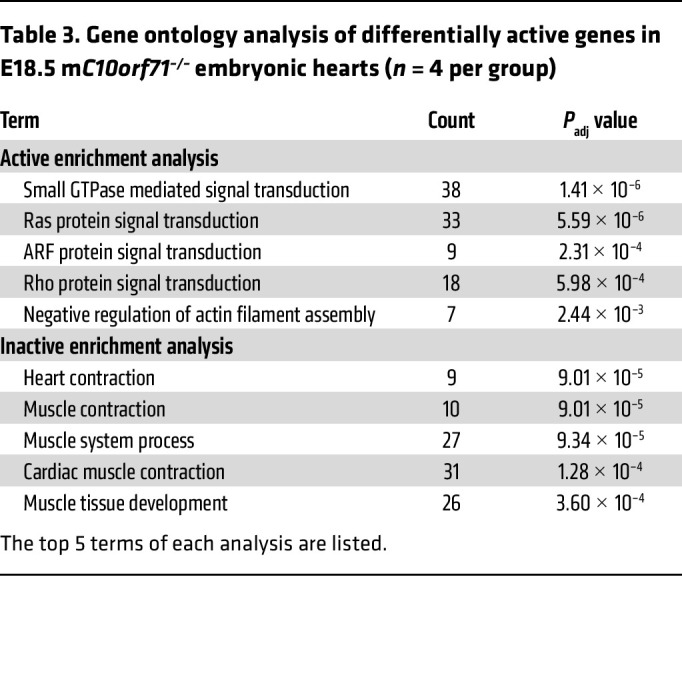
Gene ontology analysis of differentially active genes in E18.5 m*C10orf71^–/–^* embryonic hearts (*n* = 4 per group)

**Table 2:**
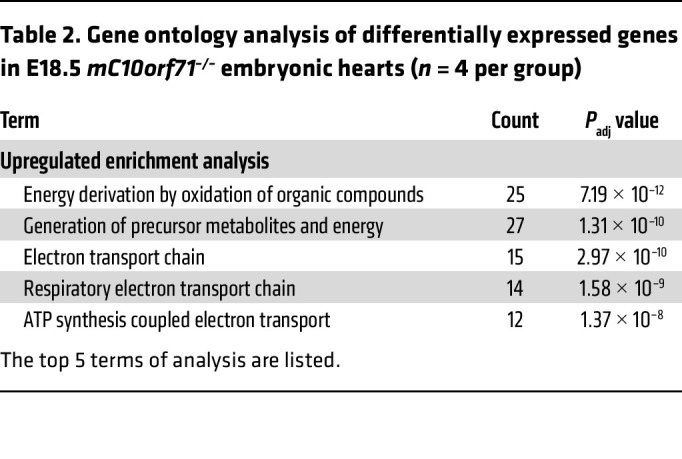
Gene ontology analysis of differentially expressed genes in E18.5 *mC10orf71^–/–^* embryonic hearts (*n* = 4 per group)

**Table 1:**
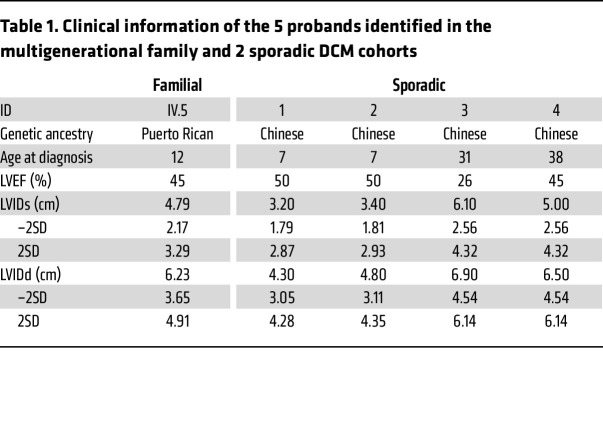
Clinical information of the 5 probands identified in the multigenerational family and 2 sporadic DCM cohorts

## Version 1 06/17/2024

Electronic publication
